# Longevity of Polymer-Infiltrated Ceramic Network and Zirconia-Reinforced Lithium Silicate Restorations: A Systematic Review and Meta-Analysis

**DOI:** 10.3390/ma14175058

**Published:** 2021-09-03

**Authors:** William Banh, Jared Hughes, Aaron Sia, David C. H. Chien, Santosh K. Tadakamadla, Carlos M. Figueredo, Khaled E. Ahmed

**Affiliations:** 1 School of Medicine and Dentistry, Griffith University, Gold Coast, QLD 4222, Australia; william.banh@alumni.griffithuni.edu.au (W.B.); drjaredhughes@gmail.com (J.H.); aaronsia97@gmail.com (A.S.); david.chien@hotmail.com.au (D.C.H.C.); s.tadakamadla@griffith.edu.au (S.K.T.); c.dasilvafigueredo@griffith.edu.au (C.M.F.); 2 Menzies Health Institute Queensland, Griffith University, Gold Coast, QLD 4222, Australia

**Keywords:** ceramic, PICN, ZLS, crown, indirect restoration

## Abstract

The purpose of this study was to systematically review the existing literature to assess the clinical survival and success of PICN and ZLS indirect restorations as the clinical evidence for them remains lacking. PubMed, SCOPUS, Embase, Cochrane Library, Web of Science, LILACs, and SciElo databases were searched from 1 January 2000 to 1 February 2021. Clinical trials and cohort studies published in English were included while case studies, case series, and in vitro studies were excluded. Results were analyzed qualitatively and a meta-analysis using a random effects model was performed. A strength of recommendation taxonomy (SORT) analysis was conducted and risk of bias (RoB) was assessed using the Newcastle–Ottawa scale and Cochrane RoB tools. An electronic search through the databases yielded 2454 articles, of which 825 remained after duplicate removal. Five studies investigating PICN and four investigating ZLS indirect restorations remained after assessing for eligibility. The overall survival rate of PICN over 1 year was 99.6% and 99.2% over 2 years. The overall survival rate of ZLS over 1 year was 99%. The main mode of failure for both materials was catastrophic fracture. One study had a high RoB, four had a moderate RoB, and four had a low RoB. Both materials demonstrated moderate strength of recommendation at a level 2 evidence for all studies based on SORT analysis. PICN and ZLS show promising short-term clinical performance as full and partial coverage indirect restorations, but longer follow-up studies are required to confirm their long-term performance.

## 1. Introduction

The exponential development of computer-aided design/computer-aided manufacturing (CAD/CAM) in dentistry has led to an increased conversion to a digital workflow and an uptake in the use of CAD/CAM materials [1]. Polymer-infiltrated ceramic networks (PICN) and zirconia-reinforced lithium silicate (ZLS) are two CAD/CAM materials that have been recently introduced into the dental market [2]. Structurally, PICN is a hybrid material comprised of a major ceramic phase infiltrated with a minor resin phase, formulating an interconnected ceramic and resin network that possesses properties of both ceramic and resin materials [3]. Scanning electron microscope (SEM) imaging and energy dispersive X-ray spectroscopy (EDS) analysis of PICN specimens revealed that the ceramic phase consists of leucite and a minor phase of zirconia [3]. The presence of micro-cracks between the ceramic and polymer phase were also observed, which could potentially reduce its mechanical properties [4]. On the other hand, ZLS is a glass ceramic with the addition of 10% zirconia by weight [5] that combines properties of both polycrystalline and glass ceramics [6]. SEM imaging of ZLS demonstrates a microstructure containing crystals that are much smaller than lithium disilicate (L.D.), which the manufacturer claims to ensure good milling and polishing characteristics of the material compared to L.D. [5,7]. According to the manufacturers, PICN is indicated for posterior, aesthetic, and implant-supported crowns and veneers [8], while ZLS is indicated for anterior and posterior crowns, veneers, inlays, and onlays [5].

In vitro testing of PICN identified that PICN had superior mechanical properties compared to feldspathic ceramics (F.C.) that remain limited when compared to L.D. On the other hand, ZLS showed superior mechanical properties compared to L.D. and PICN. The three-point flexural strength of PICN and ZLS ranged from 124 MPa to 202.1 MPa [9,10,11,12,13] and 443.64 MPa to 510 MPa, respectively [13,14,15,16], while the fracture toughness ranged from 0.86 MPam^1/2^ to 1.4 MPam^1/2^ and 2.31 MPam^1/2^ to 4.7 MPam^1/2^, respectively [7,12,15,17,18,19]. The fatigue strength of PICN and ZLS was shown to be 81.9 MPa and 152.1 MPa, respectively [20], where both materials had a lower fatigue strength than L.D. but higher than F.C. [20]. Despite this, PICN appears to be more advantageous in its higher Weibull modulus and elastic modulus. The reported Weibull modulus for PICN and ZLS ranges from 18 to 22.8 [10,18,21,22] and 5.5 to 13 [7,15,16,18], respectively, while L.D. ranges between 4.91 and 12.8 [7,9,16,18,22]. This indicates a higher structural reliability and homogeneity in PICN compared to L.D. and ZLS. The elastic modulus of PICN is reported to range from 21.5 GPa to 37.5 GPa [3,7,10,13,23], which approximates that of human dentine [24] when compared to other ceramics such as L.D. and ZLS, allowing for better stress distribution, reduced flexural damage and fatigue crack propagation [25]. The elastic modulus of ZLS is reported to range from 65.6 GPa to 70.44 GPa [7,13,15], which is higher than L.D. (60.61 GPa–63.9 GPa) [7,13,15], indicating a stiffer material. In terms of surface mechanical properties, PICN had a lower Vicker’s hardness of 1.7 GPa to 2.35 GPa [9,11,12], which is lower than ZLS (6.53 GPa–7.6 GPa) [15,19] and L.D. (5.45 GPa) [15]. Although a lower hardness has been shown to be a reliable predictor of reduced wear of enamel for resin materials [26], it does not seem to correlate well with wear properties in ceramic materials [27]. This is reflected in conflicting results of two-body abrasive wear in two studies in which PICN showed no significant difference in wear compared to the enamel on enamel control in one study [28], but demonstrated the most wear on an antagonist tooth when compared to L.D. and resin nano-ceramics (R.N.C.) in the other [22]. In terms of optical properties, PICN has acceptable optical properties with a translucency parameter of 16 but fared much worse compared to L.D and ZLS which had translucency parameters of 26 and 31, respectively [16]. This shows that ZLS may have better optical qualities compared to L.D. as translucency parameter is a critical property in recreating aesthetic restorations [16]. These unique properties of both ZLS and PICN can present them as possible management alternatives in challenging clinical situations such as tooth wear patients where the use of other ceramic indirect restorations or composite resin direct restorations come with inherent limitations. For anterior and posterior tooth wear rehabilitation, ZLS appears to offer a combination of improved mechanical and optical properties and improved surface roughness, when compared to F.C and L.D. Similarly, PICN offers major advantages for restoration of posterior teeth in tooth wear patients, especially when compared to direct composite restorations in molars which are considered medium-term management modalities that can be time-consuming and technique-sensitive [29]. These advantages relate to PICN’s satisfactory optical properties, an elastic modulus that resembles that of dentine, and a streamlined digital workflow that does not require firing and can be chairside fabricated.

Although in vitro testing of ZLS and PICN may provide an initial insight of their mechanical and optical properties, but has inherent limitations given that they are performed under controlled conditions that might not replicate clinical presentation using tests that may not be standardized, resulting in differing results that are challenging to compare [30]. Furthermore, in vitro data are not always realised in clinical studies [30]. To the best of the authors’ knowledge, there is currently no systematic review specifically investigating the survival rates of PICN and ZLS as novel CAD/CAM materials; therefore, this review will aid clinicians in the material selection process. Accordingly, the aim of this study was to investigate the clinical performance of PICN and ZLS indirect restorations through systematically reviewing the existing literature and answer the PICO question: what are the clinical survival and success rates of PICN and ZLS crowns used as indirect restorations in adult patients?

## 2. Materials and Methods

### 2.1. Protocol and Registration

The study protocol was registered in the International Prospective Register of Systematic Reviews (CRD42020195749).

### 2.2. Eligibility Criteria

All clinical (randomised and quasi) trials, as well as cohort studies, with a minimum follow up period of 6 months on adult patients were considered. Case studies, case series, and in vitro studies were excluded.

### 2.3. Information Sources

Seven electronic databases (MEDLINE, EMBASE, Scopus, Cochrane Database, Web of Science, LILACs, and SciELO) were searched to identify relevant articles for the review. The reference lists of the articles considered for full-text review were also screened by hand for any additional trials that may have been eligible for review. The search was restricted to include the articles published between 1 January 2000 and 10 June 2020. An updated search was conducted on 1 February 2021. The search was also limited to English language articles.

### 2.4. Search Strategy and Study Selection

The search strategy was peer-reviewed using the peer-review of electronic search strategies guideline (PRESS) [31] and mapped to all the different search databases. The search strategy used in PubMed is presented in Table 1.

Study eligibility was performed individually and independently by two investigators (D.C. and A.S.). Any disagreements were resolved by a third investigator (K.A.). Records retrieved from the search strategy were initially screened through title and abstract and full-text publication were reviewed if necessary to discern if they were relevant to the study. After initial screening, full-text analysis was performed to assess for eligibility based on the eligibility criteria.

### 2.5. Data Extraction

Data extraction was performed individually and independently by 2 investigators (W.B. and J.H.) and tabulated in a Word document using pre-determined data extraction charts. Disagreements were resolved by a third investigator (K.A.).

Data on variables which were extracted from included studies, if available, consisted of: author, year of publication, type of study, sample size (patient and number of restorations), clinical setting, age (mean and range), sex, restoration material and design, the reason for restoration, bonding/luting system, follow-up period, location (anterior or posterior), number and methods of failure, the method and type/criteria of clinical assessment, number of evaluators, definition of failure and success and survival, success and/or failure rates.

### 2.6. Risk of Bias in Individual Studies

Randomised controlled trials (RCT) were assessed for risk of bias using the revised Cochrane risk of bias tool [32]. Cohort studies were assessed using the Newcastle–Ottawa Scale (NOS) for selection and outcome [33]. Comparability and selection of the non-exposed cohort were omitted in the NOS as they were not applicable to the selected studies. Studies with 6 stars were rated as low RoB, 5 stars as moderate RoB, and 4 stars as high RoB. RCTs were assigned a low, medium, or high risk of bias using the risk of bias template and the suggested algorithm for risk of bias in the revised Cochrane risk of bias tool [32]. Risk of bias was evaluated independently by 2 investigators (W.B. and J.H.). Any disagreements were resolved by a third investigator (K.A.).

### 2.7. Methods of Analysis

A descriptive qualitative analysis of the included studies was conducted. Meta-analyses were performed to analyse the overall survival rates of PICN (five studies) and ZLS (four studies) restorations. Single-arm meta-analyses were performed using the fixed-effects model and presented as forest plots. A fixed effects model was used as the heterogeneity between the studies was low, and Open Meta-Analyst statistical software was used for this purpose [34]. The survival rate, expressed as a proportion of crowns surviving at the follow-up, was the summary estimate used along with 95% confidence intervals. Separate forest plots were constructed for ZLS and PICN indirect restorations, all ZLS and PICN studies reported survival rates at 1 and 2 years follow-up, respectively. Furthermore, a subgroup analysis was performed for PICN studies based on the restoration design, which included single full crowns and partial coverage restorations to assess for differences in survival. Heterogeneity of the studies was assessed using I^2^, with I^2^ < 25% being considered as low heterogeneity and <50% as moderate heterogeneity [35].

An additional assessment was performed using the Strength of Recommendation Taxonomy (SORT) to evaluate the level of evidence of individual studies and the strength of recommendation [36].

## 3. Results

### 3.1. Study Selection

**Figure 1:**
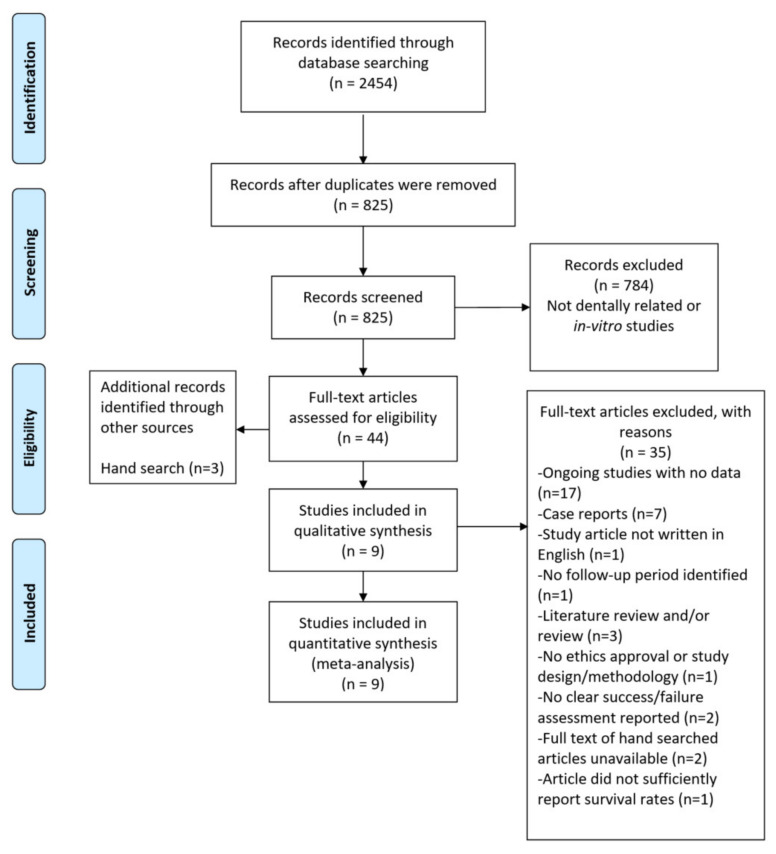
Flow diagram of the study selection process.

The initial search through all electronic databases yielded 2454 articles (Figure 1). After the removal of duplicate articles, 825 articles remained. After screening the titles and abstracts, 784 were excluded. The remaining 41 full-text articles were assessed for eligibility based on the inclusion criteria and an additional 3 articles were found hand-searching the included articles. Thirty-five of the included articles were excluded with reasons given for their exclusion shown in Figure 1. The nine remaining studies were included in qualitative and quantitative data synthesis [37,38,39,40,41,42,43,44,45]. During an updated search on 1 February 2021, only one additional clinical study was found, but was not included as it was a case series.

### 3.2. Descriptive Analysis

#### 3.2.1. Background Characteristics of the Included Studies

Of the nine included studies, five investigated PICN [37,38,40,43,44] and four investigated ZLS (Table 2) [39,41,42,45]. Studies investigating PICN were published from 2016 to 2020 and had follow up periods ranging from 2 to 3 years, of which four had a prospective study design [38,40,43,44] and one retrospective design [37]. In total, there were 433 PICN indirect restorations assessed at the end of the follow-up periods. Of the five studies, four reported some degree of restoration attrition ranging from 6.9% [38] to 23.3% [43]. Four of the studies were performed in a university setting [38,43,44,45], and only one was performed in a private practice setting [37]. Two studies excluded patients with temporomandibular disorders (TMD), parafunctional habits, alcohol, drug abuse, or life threatening diseases [43,44], and one study excluded patients with cracks or fracture lines on the abutment tooth [38]. Oudkerk et al. [40] excluded patients with poor oral hygiene, periodontal disease, pre-existing crowns, bridges or implants, Parkinson’s disease or TMD, or were smokers. On the other hand, Chirumamilla et al. [37] did not explicitly report any exclusion criteria. Only Lu et al. [38] had a comparative cohort which compared feldspathic with PICN.

Studies investigating ZLS were published in a similar time frame from 2017 to 2020. The follow-up period for the studies ranged from 1 to 3 years, with two studies presenting only a 1 year follow-up period [39,45]. Of the studies investigating ZLS, three were prospective study designs [41,42,45] and one was a double-blinded randomised controlled split-mouth design [39]. A total of 230 restorations were assessed at the end of the follow up periods with restoration drop-out rates ranging from 2.2% [41] to 9.75% [45]. Of the four studies, three were performed in a university setting [39,41,45] and one in a private practice setting [42]. All studies involving ZLS excluded patients with bruxism. Two studies had additional exclusion criteria of deep subgingival margins, untreated periodontal disease, an oro-vestibular defect size of <50% of the tooth cusp distance, and patients less than 18 years old [41,42]. Nassar et al. [39] also excluded patients with severe systemic disorders, smokers, patients with xerostomia, and teeth that were non-vital, endodontically treated, mobile, or periodontally affected. Zimmermann et al. [45] also excluded patients with TMD and additionally teeth that needed direct/indirect capping prior to treatment due to their study focusing on inlay/partial crown restorations. Only Nassar et al. [39] had a lithium silicate partial coverage crown control group.

#### 3.2.2. Restoration Characteristics

Three of the five studies investigating PICN were limited to the posterior teeth only [37,38,43], while the other two studies included both anterior and posterior teeth [40,44]. Of the studies that included anterior teeth, no distinction between the survival rates of anterior and posterior teeth was reported. All studies which investigated ZLS were limited to the posterior region only [39,41,42,45].

There was a large variety with regard to the type and design of the restorations placed in the studies investigating PICN. Two studies used a single-unit full-coverage crown [37,44], one study used onlays [38], and one study used inlay and partial coverage crown designs [43]. Oudkerk et al. [40] had the widest variety of restorations designs within the study, including palatal veneers, posterior occlusal tabletops, and veneerlays, owing to the “one-step no-prep” approach. Conversely, studies investigating ZLS had a more consistent restoration design across the board, with all of them having a partial crown design [39,41,42,45].

Three of the five studies investigating PICN used a dual cure resin cement [38,43,44]. One study [37] used both resin-modified glass ionomer cement (RMGIC) and dual cure resin cement with no significant difference in survival probability between the two types of cement. Oudkerk et al. [40] cemented all PICN restorations with a light-cured self-etch resin cement.

Two of the four studies investigating ZLS used a dual cure resin cement to seat all their restorations [39,45]. Under a randomised process, the remaining two studies [41,42] demonstrated no statistically significant difference in survival between dual curing resin cement and dual cure self-adhesive cement over 3 years.

#### 3.2.3. Outcome (Survival/Success) Assessment

The definition of survival varied amongst the studies which investigated PICN [37,38,40,43,44]. One study [37] classified crown fractures, crown chipping, crown debonding, and endodontic treatment of abutment tooth or internal crack of abutment tooth as failures. Two studies [43,44] used a similar definition but also included unacceptable marginal discolouration and adaptation, secondary caries, crown chipping, endodontic treatment, and cracks of the abutment tooth as failures. Two studies did not explicitly define criteria for survival [38,40]. In terms of success, two studies [43,44] defined relative failures as minimal cohesive fractures, minor cracks that are clinically acceptable, minor marginal stains and minor marginal deviations in marginal fit. One study did not explicitly define a criteria [38] and two studies did not investigate success [37,38]. Similarly, there were discrepancies between studies investigating ZLS regarding their definition of survival. Two studies [41,42] defined survival as restorations that remained in situ without signs of total loss such as clinically unacceptable ceramic fracture or biological events such as caries, tooth fracture, or periodontal disease which required replacing of the tooth or restoration. Another study [39] had a similar definition, but also included the loss of retention as an absolute failure. One study [45] defined failure as subcategories 4 and 5 (clinically not sufficient but repairable and clinically inacceptable respectively) in the modified FDI criteria. Two studies defined success as any restoration that remained unchanged and functional in situ and intervention-free [41,42], one study did not state a clear definition for success [45] and another did not investigate success [39].

All studies investigating PICN assessed restorations clinically at their respective follow-up periods, with one study using the modified California Dental Associated criteria (CDA) [37], three using the modified United States Public Health Services (USPHS) criteria [38,43,44], and one using the World Dental Federation criteria (FDI) [40]. Likewise, all studies investigating ZLS assessed their restorations clinically at their respective follow-up periods with three studies using the modified USPHS criteria [39,41,42] and one using the modified FDI criteria [45].

#### 3.2.4. Modes of Failure

Due to differences in the definition of survival, debonding was reclassed as absolute failures for the purpose maintaining consistency of reporting.

In studies investigating PICN, the most common method of absolute failure was due to a catastrophic fracture to the material (*n* = 7/433), followed by abutment cracks or fractures (*n* = 2/433) and debonding of the restoration (*n* = 2/433). There was also a biological failure due to secondary decay with subsequent debonding of the restoration (*n* = 1/433). In terms of relative failures, the only reported method of failure was due to minor chipping of the restoration (*n* = 16/433).

Similarly, in studies investigating ZLS, the most common method of absolute failure was also due to catastrophic bulk fractures of the material (*n* = 5/230). This was followed by debonding (*n* = 2/230), and fracture of the abutment tooth (*n* = 1/230). The only reported relative failure was the loss of vitality of an abutment tooth (*n* = 1/230), which was subsequently endodontically treated.

### 3.3. Quantitative Analysis

#### Survival and Success

**Figure 2:**
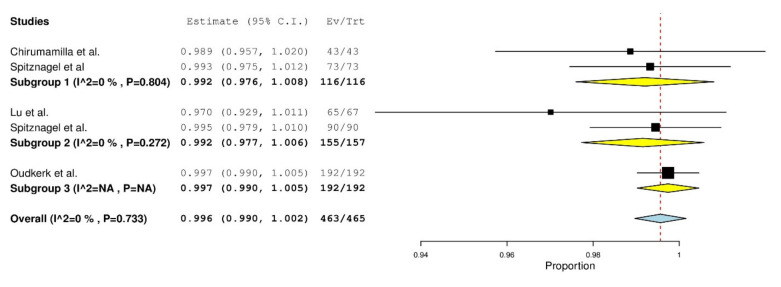
One-year survival forest plot for PICN.

**Figure 3:**
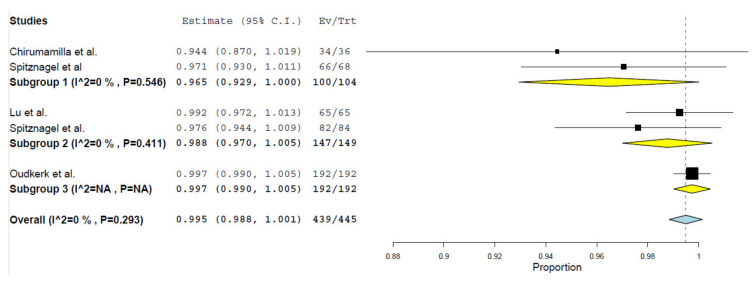
Two-year survival forest plot for PICN.

**Figure 4:**
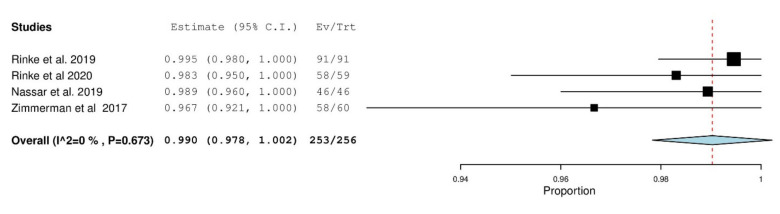
One-year survival forest plot for ZLS.

After quantitative analysis, the 1 year overall survival rate of PICN was determined to be 99.6% (*p* < 0.001) and 99.5% (*p* < 0.001) over 2 years demonstrated in Figure 2 and Figure 3 respectively. The 1 year overall survival rate of ZLS was 99% (*p* < 0.001) as demonstrated in Figure 4. The length of the survival rates analysed were limited by the study with the shortest follow-up durations. Due to the differences in classifying failures of restorations, the survival data from Rinke et al. [42] was manually recalibrated such that debonding was reclassified as an absolute failure. Recalibration was unable to be conducted for Oudkerk et al. [40] due to insufficient information reported in the study to accurately recalibrate the survival data. There is a low amount of heterogeneity amongst studies investigating PICN and ZLS given the I^2^ value of 0% [35].

A subgroup analysis of the survival of PICN restorations in relation to the design of the restorations was also conducted. Over a 1 year follow-up period, both single full coverage (subgroup 1) and partial coverage (subgroup 2) PICN crowns had an overall survival rate of 99.2% (*p* < 0.001). A combination of conservative restorations including palatal veneers, veneerlays and occlusal tabletops using a “one-step no-prep” approach (subgroup 3) had a survival rate of 99.7%. In the 2 year follow up, single full coverage crowns had an overall survival rate of 96.5% (*p* < 0.001), while partial coverage restorations had an overall survival rate of 98.8% (*p* < 0.001) and the “one-step no-prep” approach had an overall survival rate of 99.7%. Heterogeneity was low (I^2^ = 0) between the studies in both the subgroups for 1 and 2 year survival. A similar analysis was not conducted for ZLS as all studies had a partial coverage restoration (inlay or onlay).

An analysis on the success rate for both PICN and ZLS was planned but not completed as not all studies reported a success rate as well as inconsistent or obscure criteria for measuring success was reported. Therefore, it was deemed inappropriate to conduct a quantitative analysis with the remaining studies.

### 3.4. Risk of Bias within Studies

The Newcastle–Ottawa scale was used to assess eight studies which were cohort studies [37,38,40,41,42,43,44,45]. One randomised control trial was assessed with the revised Cochrane Risk of Bias tool [39]. One study was at a high risk of bias [38], four were considered a moderate risk of bias [37,40,43,44], and four were considered a low risk of bias (Table 3 and Table 4) [39,41,42,45].

### 3.5. Strength of Recommendation

All nine included studies were graded as level 2 for evidence of individual studies and an overall moderate strength of recommendation for both PICN and ZLS due to a combination of short follow-up times, limited sample sizes, lack of independent evaluators, and clinical heterogeneity of the studies limited the level of evidence of all studies and the strength of recommendation of the two materials.

## 4. Discussion

The aim of this systematic review was to investigate the performance of two CAD/CAM materials, PICN and ZLS, as indirect restorations. Survival at 1 year for PICN and ZLS was >99% at 1 year, and >99% at 2 years for PICN. Among PICN restorations, full coverage restorations demonstrated higher survival than partial coverage. A quantitative analysis was not carried out for the success rates of each material due to limited reporting and varying definitions of success employed in the included studies. To the best of the authors’ knowledge, this is the first systematic review investigating the clinical survival and success of PICN and ZLS CAD/CAM materials. Previous reviews investigating the performance of indirect restorations were not able to discern the performance of individual CAD/CAM indirect restorations due to the heterogeneity of included studies assessing different materials with varying compositions, and instead reported overall survival rates with no meta-analysis specific to each material [46,47].

The most common methods of failure for both PICN and ZLS were catastrophic and bulk fractures of the material followed by debonding of the restoration. Similarly, reviews investigating ceramic, composite, and hybrid restorations [48,49,50], all report ceramic fractures as the main mode of failure. Therefore, it seems that PICN and ZLS are no exceptions to this common mode of failure. For relative failures, studies investigating PICN reported minor chipping of the ceramic as the most common occurrence while ZLS reported no incidences of chipping. In vitro testing of edge chipping resistance by Argyrou et al. [10] also indicated that PICN has lower edge toughness than resin nanoceramics and feldspathic ceramics. However, it is worth noting that a majority of the incidences of chipping in PICN occurred in the Oudkerk et al. [40] study where all patients reported parafunctional habits in the form of grinding or clenching in-conjunction with the study’s limited sample size (seven participants). A retrospective study by Beier et al. [51] involving 1335 silicate glass-ceramic crowns, inlays, onlays and veneers placed between 1987 and 2009 demonstrated that there was a 2.3 times greater risk of failure in patients presenting parafunctional habits. A systematic review on the clinical performance of partial and full coverage CAD/CAM restoration by Al-Haj Husain et al. [47] also reported ceramic chipping as the most common technical complication with no statistical difference amongst hybrid polymer and ceramic materials, which also aligns with results from Vagropoulou et al. [52]. Biological complications were less common than technical failures in both types of ceramics. One incidence of secondary caries and one incidence of crack propagation in the abutment tooth were associated with PICN [37] and one incidence of loss of vitality was reported in ZLS [42]. Likewise, the most reported biological failure was caries and loss of pulp vitality in the review by Al-Haj Husain et al. [47] From the included studies, there were no reported statistical differences in survival between RMGIC and dual cure self-adhesive resin cement in PICN [38] and dual cure resin cement and self-adhesive resin cement in ZLS [42].

In terms of survival, PICN appears to perform slightly worse than more established ceramic materials such as L.D. and monolithic zirconia. A systematic review by Pieger et al. [53] reveals that the 2 year cumulative survival rate for L.D. single crowns was 100% which is higher compared to a 96.5% overall survival of PICN single full-coverage crowns reported in the current study. Similarly, a study by Worni et al. [54] demonstrated a 100% survival rate of monolithic zirconia crowns over a median follow up period of 23.8 months. Lu et al. [38] reported a 97% survival for PICN and 90.7% for feldspathic onlays but reported no statistical differences in survival between the two materials over 3 years. The differences in performance seem to be consistent with the results of in vitro studies performed on PICN, L.D., F.C., and zirconia [7,9,11,12,13]. In regard to how resin matrix ceramics in general fare against other CAD/CAM materials, a review investigating the clinical performance of CAD/CAM restorations reports an estimated failure rate of 3.85% per 100 in resin-matrix ceramics compared to 1.79% in glass-matrix ceramics and 4.07% in polycrystalline ceramics [46], which is consistent with Al-Haj Husain et al. [47]. The results of the present study also agree with the systematic review by Al-Haj Husain et al. [47] which reported that a similar overall survival rate of partial and full restorations made of hybrid polymer ceramics as 99% in 24 months.

The initial survival rate of ZLS appears to be promising and comparable to L.D. and zirconia ceramics; however, the results of the present study are limited to 1 year and a partial coverage design. In comparison to L.D., in which ZLS demonstrated better in vitro mechanical properties, both materials seem to be similar in terms of short-term survival [15,17]. A RCT of posterior L.D. partial crowns by Ferrari et al. [55] had a 100% survival rate over 3 years, while Rinke et al. [42] reported a 99% survival in 3 years. In the RCT conducted by Nassar et al. [39], L.D. and ZLS both had a 100% survival rate over 1 year. Likewise, a prospective clinical study of monolithic CAD/CAM zirconia reported a 100% survival rate over 1 year [56]. At the present time, only limited conclusions can be drawn about how ZLS compares with other ceramic materials due to its short follow-up period of 1 year.

The current systematic review has a number of limitations. The short follow-up periods ranging from 1 to 3 years within the included studies can only provide an insight into the short-term prognosis of the restorations and may not identify failures which may emerge in trials with longer follow-up periods. Furthermore, the limited sample size of the included studies without any reported power calculations leads to potential type I and type II statistical errors within the studies that are not accounted for. As well as this, five of the nine studies included in the review were also considered either at a high or medium risk of bias which may limit the robustness of the meta-analysis conducted within this study. A single arm meta-analysis was conducted as only two among the included nine studies had a comparison [38,39]. Furthermore, there were issues with reporting in several studies with the calibration of assessors not always explicitly reported.

In terms of future investigations, longer follow-up periods are required to assess the long-term prognosis of both materials. Furthermore, a more consistent definition of survival and success, which is explicitly reported, is required to more reliably and accurately determine the survival and success rates of these restorations. Standard descriptive guidelines and methods of clinical reporting survival and success by Anusavice [57] should be considered in future studies to help reduce discrepancies in methodology and reporting and therefore reduce heterogeneity between studies. Different designs other than partial coverage restorations should also be investigated for ZLS such as full coverage crowns, given its proposed superior mechanical properties to L.D. shown through in vitro testing.

## 5. Conclusions

Based on the current systematic review, PICN and ZLS demonstrated excellent clinical performance in the short term with a moderate strength of recommendation. Nonetheless, these findings need to be interpreted with caution as longer follow-up studies are required to support the long-term performance of PICN and ZLS indirect restorations.

Further studies involving different restoration designs are required to support its use for the different indications. The current studies have mainly focused on partial coverage or full coverage crowns, but further investigations into survival and success of other restorations such as veneers or bridge designs both in the anterior and posterior regions would be beneficial. In particular, ZLS has demonstrated superior physical properties compared to LD and therefore it may be of interest to investigate whether they are more suited clinically as anterior or posterior bridges compared to LD. This may allow for more alternatives for a monolithic bridge design with good aesthetic properties. PICN has also been proposed for use as implant crowns given it has an elastic modulus similar to human dentine [24], and further investigations as to whether this makes a significant difference in the survival and success of implant restorations is necessary.

Additional parameters and patient-oriented treatment outcomes should also be investigated for both materials such as colour stability and resistance to wear, especially in challenging clinical presentations such as tooth wear. The colour stability of PICN over time may be of particular interest given that it also contains a resin component within the material [58]. The influence of different bonding systems and protocols has also briefly been investigated in some of the studies included, but further investigation is necessary to confirm these results. It may also be beneficial to see if the presence of zirconia in ZLS affects the bonding of the restoration and if chemical conditioning affects bond strength to the zironica component clinically [59].

## Figures and Tables

**Table 1:** Search strategy for PubMed.

	Search	Results
#1	picn OR picns OR enamic	291
#2	(“resin matri *” OR resin-matri *) NEAR/4 ceramic *	45
#3	(“polymer-infiltrated” OR “polymer infiltrated”) NEAR/4 ceramic *	147
#4	((picn OR picns OR enamic) OR ((“resin matri *” OR resin-matri *) AND ceramic *)) OR ((polymer-infiltrated OR “polymer infiltrated”) AND ceramic *)	364
#5	zircon* NEAR/4 (“lithium silica *” OR lithium-silica *)	123
#6	zls NOT “zimmermann laband”	101
#7	“celtra duo” OR suprinity	121
#8	((zircon* AND (“lithium silica *” OR lithium-silica *)) OR (ZLS NOT “zimmermann laband”)) OR (“celtra duo” OR suprinity)	217
#9	(((picn OR picns OR enamic) OR ((“resin matri *” OR resin-matri *) AND ceramic *)) OR ((polymer-infiltrated OR “polymer infiltrated”) AND ceramic *)) OR (((zircon * AND (“lithium silica *” OR lithium-silica *)) OR (ZLS NOT “zimmermann laband”)) OR (“celtra duo” OR suprinity))	519

Note: #4 = #1 OR #2 OR #3, #8 = #5 OR #6 OR #7, #9 = #4 OR #8, * = truncated term.

**Table 2:** Data table of all included studies.

Author/Year	Type of Study	Sample Size	Clinical Setting	Age (Mean, Range), Gender	Restoration (Material, Design)	Reason for Restoration	Bonding or Luting System	Follow up Period	Location (Anterior/ Posterior)	Number and Mode of Failure	Method, Clinical Assessment Details	Definition of Survival/ Success	Survival/Failure/ Success Rates
Chirumamilla et al. (2016)	R	35 patients 45 restorations Follow up 29 patients 36 restorations	PrvP	(58, 26–85). 80% female, 20% male	PICN (VITA Enamic) Single unit full coverage crown	N.R	FujiCem (RMGIC) and Breeze or G-Cem LinkAce (Dual cure self-adhesive resin cement)	2 years	Posterior 35 molars 10 premolars 20 maxillary 25 mandibular Follow up N.R	2 absolute failures Secondary decay + debonding (*n* = 1), extension of pre-existing crack on abutment tooth requiring extraction (*n* = 1)	Clinical assessment Modified California Dental Association criteria 1 year evaluation: 1 non-independent evaluator 2 year evaluation: 1 non-independent evaluator, 1 independent evaluator	Failures (survival): crown fracture, crown chipping, crown debonding, endodontic treatment or internal crack of abutment	96.8% survival (Dual cure resin cement) 92.9% survival (RMGIC) No SSD between cements
Lu et al. (2018)	P	93 patients 101 restorations 67 Vita Enamic 34 Vitablocs Mark II Follow up 91 patients 94 restorations 65 VITA Enamic 29 VITAblocs Mark II	Uni	(37.66, 18–71). 61% female, 39% male	PICN (VITA Enamic) and Feldspathic (VITA blocs Mark II) Onlay	Restorations for endodontically treated posterior teeth	NEXUS (Dual cure resin cement)	3 years	Posterior 80 molars 21 premolars Follow up N.R	5 absolute failures PICN Debonding (*n* = 1), tooth fracture (*n* = 1) Feldspathic debonding (*n* = 2), ceramic fracture (*n* = 1)	Clinical assessment USPHS criteria 2 non-independent evaluators	Failures (survival): criteria for failure not explicitly reported Debonding of restoration, ceramic fracture, tooth fracture	97% survival PICN 90.7% survival Feldspathic No SSD in survival
Rinke et al. (2019)	P	71 patients 92 restorations Follow up 69 patients 88 restorations	PrvP	(48.9, N.R). 65% female, 35% male	ZLS (Celtra Duo) Partial crown restorations	N.R	Group A: Variolink (Dual cure resin cement) Group B: Calibra Cementation System (Self- adhesive resin cement)	3 years	Posterior 74 molars 18 premolar Location not Reported Follow up 36 maxillary 52 mandibular 71 molar 17 premolar	2 absolute failures Fracture of abutment (*n* = 1) Catastrophic fracture of ceramic (*n* = 1) 1 relative failure Loss of vitality of abutment (*n* = 1)	Clinical assessment Modified USPHS criteria 1 independent calibrated evaluator	Survival: restoration remained in situ without signs of total loss (clinically unacceptable ceramic fracture of the restoration or a biological event (caries, tooth fracture or periodontal disease) requiring complete replacement of the restoration or tooth extraction Success: reconstruction remained unchanged and functional in situ and intervention free during entire observational period	99% Survival (overall) 98% success (overall) Group A: 98% survival (3 year) Group B: 100% survival (3 year) No SSD between group A and B
Rinke et al. (2020)	P	45 patients 61 restorations Follow up 44 patients 59 restorations	Uni	(50.7, N.R). 61% female, 39% male	ZLS (VITA Suprinity) Partial crown restorations	Teeth with indications for full covering restorations using ZLS ceramic material	Variolink (Dual-curing resin cement) OR RelyX Unicem (Dual cure self-adhesive cement)	2 years	N.R Follow up 36 molars 23 premolars 34 maxillary 25 mandibular	2 absolute failures Fracture of ceramic (*n* = 2) 2 clinical interventions/relative failures Loss of retention of PCR (*n* = 2)	Clinical assessment Modified USPHS criteria 1 independent calibrated evaluator	Survival: restoration remained in situ without signs of total loss (clinically unacceptable ceramic fracture or a biological event (caries, tooth fracture or periodontal disease) requiring complete replacement of the restoration or tooth extraction Success: reconstruction remained unchanged and functional in situ and intervention free during follow-up	97% survival (overall) 93% success (overall) No SSD between thickness of material No SSD between cements
Nassar et al. (2019)	RCT	14 patients 46 restorations 23 VITA Suprinity 23 IPS e.max Follow up No Loss of patients	Uni	(N.R, N.R). 57% female, 43% male	ZLS (VITA Suprinity) and lithium disilicate (IPS e.max CAD) Partial coverage restorations	N.R	Duolink (Dual cure resin cement)	1 year	Posterior 20 maxillary 26 mandibular	No failures reported	Clinical assessment Modified USPHS criteria Number of evaluators: N.R	Absolute Failure: loss of retention, fracture, crack development requiring replacement, secondary caries or endodontic complication	100% survival
Zimmermann et al. (2017)	P	41 patients 67 restorations Follow up 37 patients 60 restorations	Uni	(45, N.R). 41% female 59% male. Follow up 46% female 54% male	ZLS (Celtra Duo) Partial crowns and inlays	N.R	Dual cure resin cement (brand N.R)	1 year	Posterior 42 molars 25 premolars 38 maxillary 29 mandibular Follow up 38 molars 22 premolars 34 maxillary 26 mandibular	2 absolute failures Bulk fracture of restoration (*n* = 2)	Clinical assessment Modified FDI criteria 1 independent calibrated evaluator	Failures: modified FDI criteria subcategories 4 and 5 (clinically insufficient but repairable, clinically unacceptable) Success: N.R	3.3% failure 96.7% success
Spitznagel et al. (2020)	P	34 patients 76 restorations Follow up 27 patients 61 restorations	Uni	(52.6, N.R). 58.8% female, 41.2% male	PICN (VITA Enamic) Full coverage single crowns	Adult patients in need of full-coverage crowns	Variolink II (Dual cure resin cement)	3 years	Anterior + Posterior 28 molars 41 premolars 7 incisors 48 maxillary 28 mandibular Follow up N.R	4 absolute failures Catastrophic fracture of the restoration (*n* = 4)	Clinical assessment Modified USPHS criteria 2 independent evaluators	Absolute failures (survival): Clinically unacceptable fracture, secondary caries, debonding, Charlie classification of clinically unacceptable marginal discolouration or adaptation Relative Failures (success): clinically acceptable deteriorations such as minimal cohesive fractures, minor cracks, minor marginal staining or deviation in marginal fit (Bravo)	93.9% survival 92.7% success
Spitznagel et al. (2018)	P	47 patients 103 restorations Follow up 37 patients 79 restorations	Uni	(47.6, N.R). 66% female, 34% male	PICN (VITA Enamic) Inlays and partial coverage restorations	Patients with carious lesions, insufficient fillings, or inlay restorations	Variolink II (Dual cure resin cement)	3 years	Posterior 63 molars 40 premolars Follow up N.R	3 absolute failures Bulk fracture of restoration (*n* = 3) 4 relative failures Minimal cohesive fractures (chipping) (*n* = 4)	Clinical assessment Modified USPHS criteria 2 independent evaluators	Absolute failures (survival): Clinically unacceptable fractures requiring replacement of restoration, inacceptable marginal discolouration, marginal adaptation, and secondary caries or debonding Relative failures (Success): Minimal cohesive fractures and clinically acceptable minor cracks, minor marginal stains and minor deviations in marginal fit	96.4% survival (overall) 97.4% survival (inlay) 95.6% survival (PCR) 82.4% success (PCR) 84.8% success (inlay)
Oudkerk et al. (2019)	P	7 patients 192 restorations Follow up No loss of patients	Uni	(37.7, N.R). 14% female, 86% male	PICN (VITA Enamic HT), palatal veneer, posterior occlusal tabletops and veneerlays	Patients with severe wear of dentition with aesthetic or functional demand	Nexus XTR system (self-etch resin cement)	2 years	Anterior + posterior 58 molars 56 premolars 26 canines 52 incisors	12 relative failures Debonding of restoration (*n* = 1) Minor chipping (*n* = 12)	Clinical assessment FDI criteria 2 independent calibrated evaluators	Survival: not clearly reported. Each item on the FDI criteria is assessed on a Likert scale. Restorations scored a 5 were deemed requiring replacement Success: N.R	100% 1 year survival 100% 2 year survival 100% 1 year success 93.7% 2 year success

R = retrospective; P = prospective; Uni = university; PrvP = private practice; RCT = randomised controlled trial; SSD = statistically significant difference; ZLS = zirconia-reinforced lithium silicate; PICN = polymer-infiltrated ceramic network; N.R = not reported/unclear; USPHS = modified U.S. Public Health Service criteria; FDI = FDI World Dental Federation; PCR = partial coverage restoration; FujiCem (GC Corporation, Tokyo, Japan); Breeze (Pentron, Orange, CA, USA); G-Cem LinkAce (GC Corporation, Tokyo, Japan); NEXUS (Kerr Corporation, Orange, California, US); Variolink (Ivoclar Vivadent, Schaan, Liechtenstein); Calibra Cementation System (Dentsply Sirona, Bensheim, Germany); RelyX Unicem (3M ESPE, Seefeld, Germany); Duolink (BISCO, Schaumburg, USA); Vaiolink II (Ivoclar Vivadent, Schaan, Liechtenstein); Nexus XTR (Kerr Corporation, Orange, California, US); VITA Suprinity (Vita Zahnfabrik, Bad Säckingen, Germany); Celtra Duo (Dentsply Sirona, Bensheim, Germany); VITA Enamic (Vita Zahnfabrik, Bad Säckingen, Germany); VITAblocs Mark II (Vita Zahnfabrik, Bad Säckingen, Germany); IPS e.max CAD (Ivoclar Vivadent, Schaan, Liechtenstein.

**Table 3:** Newcastle–Ottawa risk of bias table.

Study	Selection			Outcome			
	Representativeness of exposed cohort	Ascertainment of exposure	Demonstration that outcome of interest was not present at start of study	Assessment of outcome	Was follow up long enough for outcomes to occur?	Adequacy of follow up of cohort	Out of 6 stars
Chirumamilla et al.	*	*	*	-	*	*	5
Lu et al.	-	*	*	-	*	*	4
Rinke et al. (3 year)	*	*	*	*	*	*	6
Rinke et al. (2 year)	*	*	*	*	*	*	6
Zimmermann et al.	*	*	*	*	*	*	6
Spitznagel et al. (2020)	*	*	*	*	*	-	5
Spitznagel et al. (2017)	*	*	*	*	*	-	5
Oudkerk et al.	-	*	*	*	*	*	5

Asteriks (*) = Star obtained in the Newcastle Ottawa Scale.

**Table 4:** Cochrane Risk of Bias Tool Table.

Study	Nassar et al.
Domain 1: Risk of bias arising from the randomisation process	Low
Domain 2: Risk of bias due to deviations from the intended interventions	Low
Domain 3: Missing outcome data	Low
Domain 4: Risk of bias in measurement of outcome	Low
Domain 5: Risk of bias in selection of the reported result	Low
Overall risk of bias	Low

## Data Availability

Not Applicable.
